# Supplementary figures and images for: Cell type specific allometry controls sex-differences in Drosophila body size
Source: bioRxiv. 2025 Aug 25:2025.08.25.671808. Preprint. [Version 1] doi: 10.1101/2025.08.25.671808 (PMC12407806; doi:10.1101/2025.08.25.671808)

Figure S3 - Expression Bias in Head

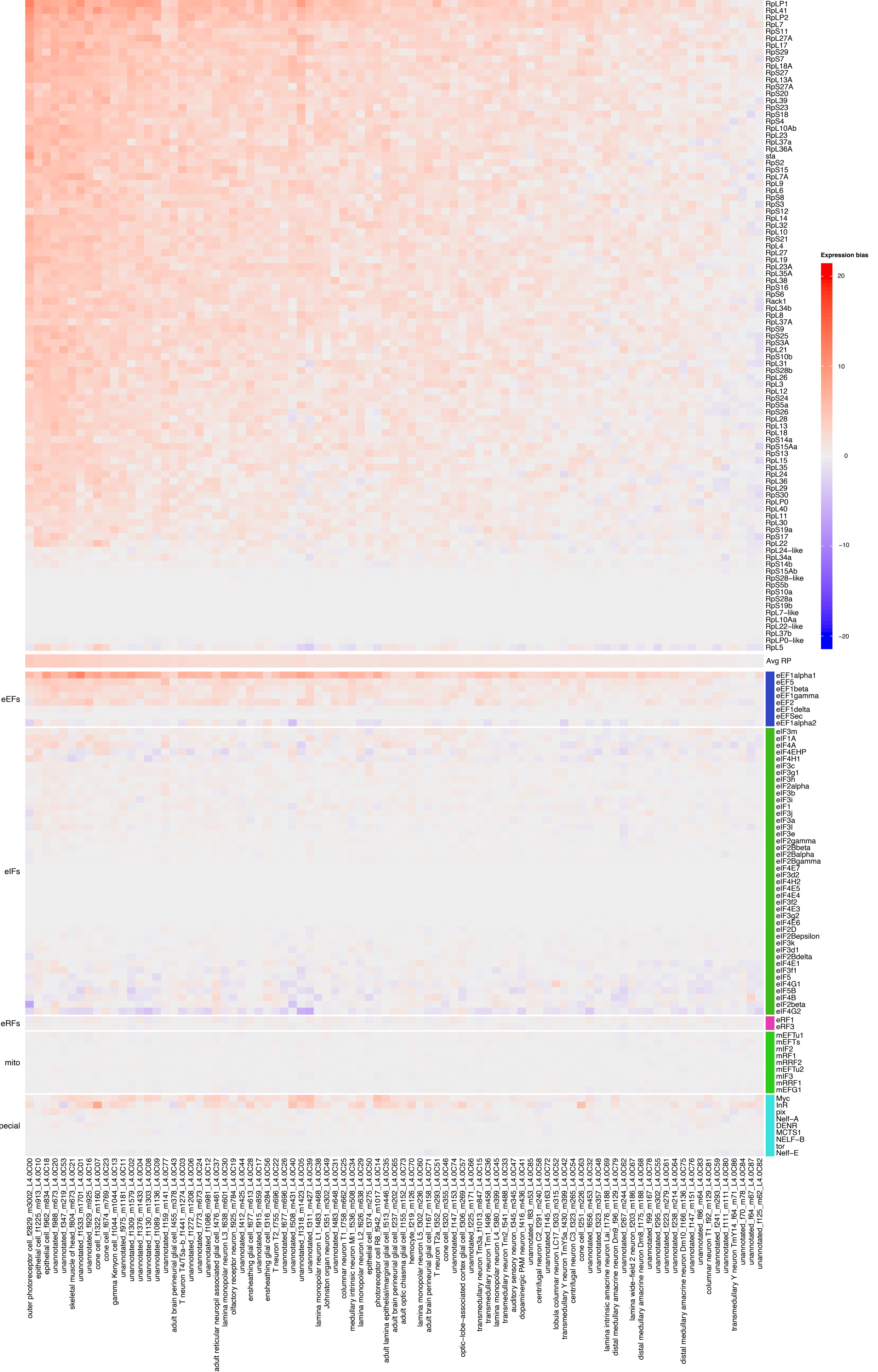

Supplement: Supplement 3 [file media-3.pdf]

## Figure S4 - Expression Bias in Body

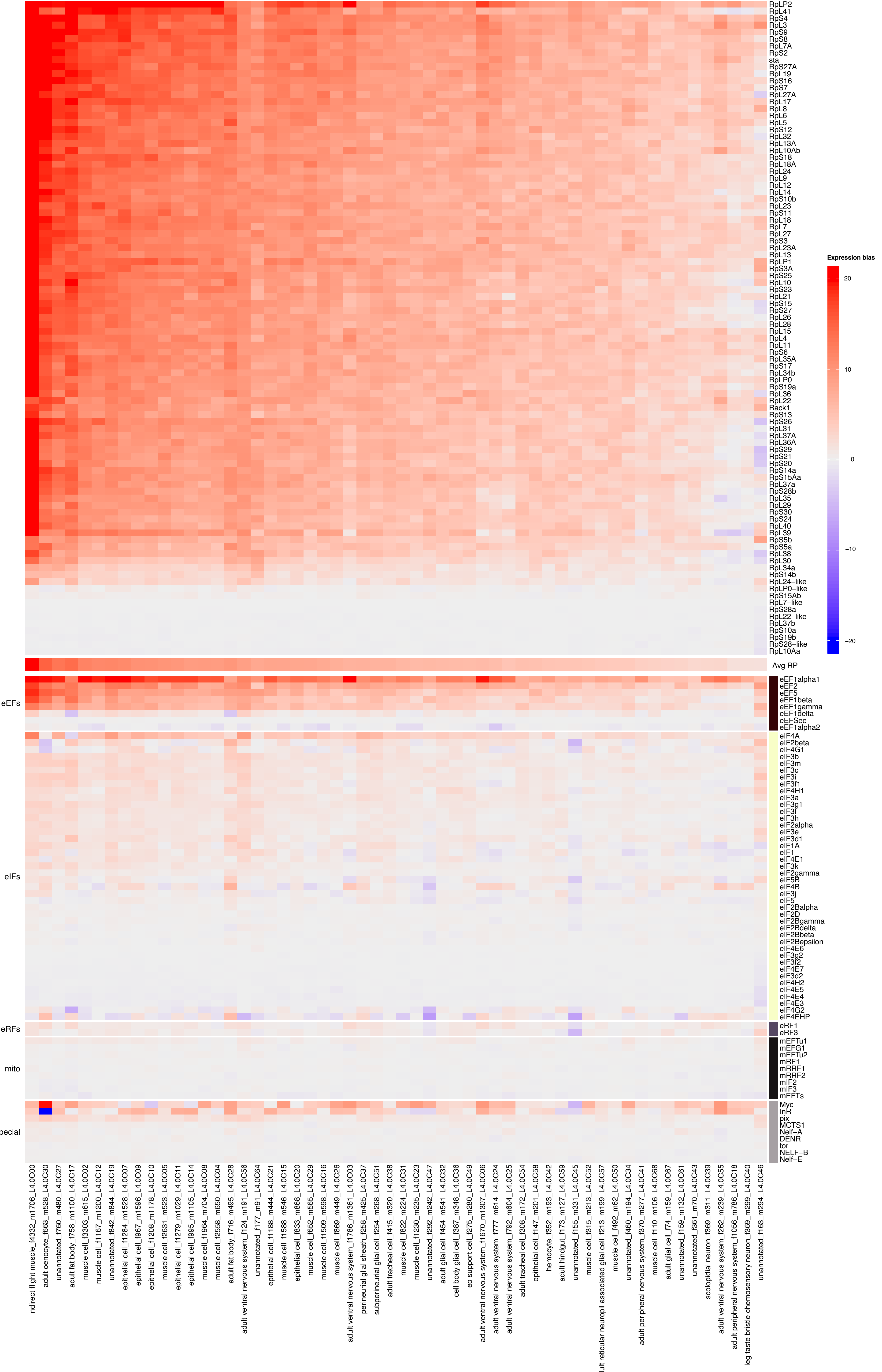

Supplement: Supplement 4 [file media-4.pdf]

Figure S5 - Expression Bias in Antenna

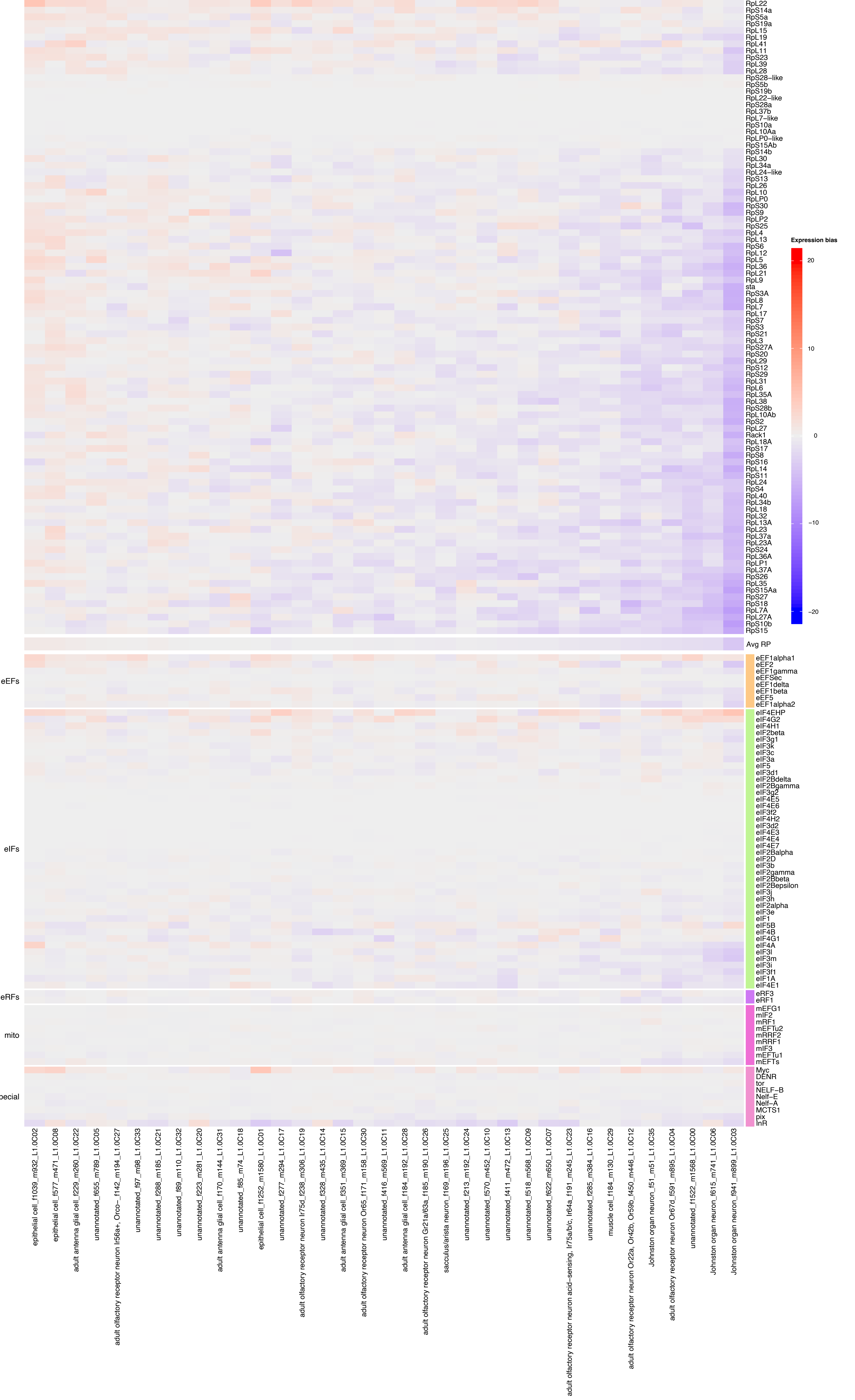

Supplement: Supplement 5 [file media-5.pdf]

Figure S6 - Expression Bias in Body Wall

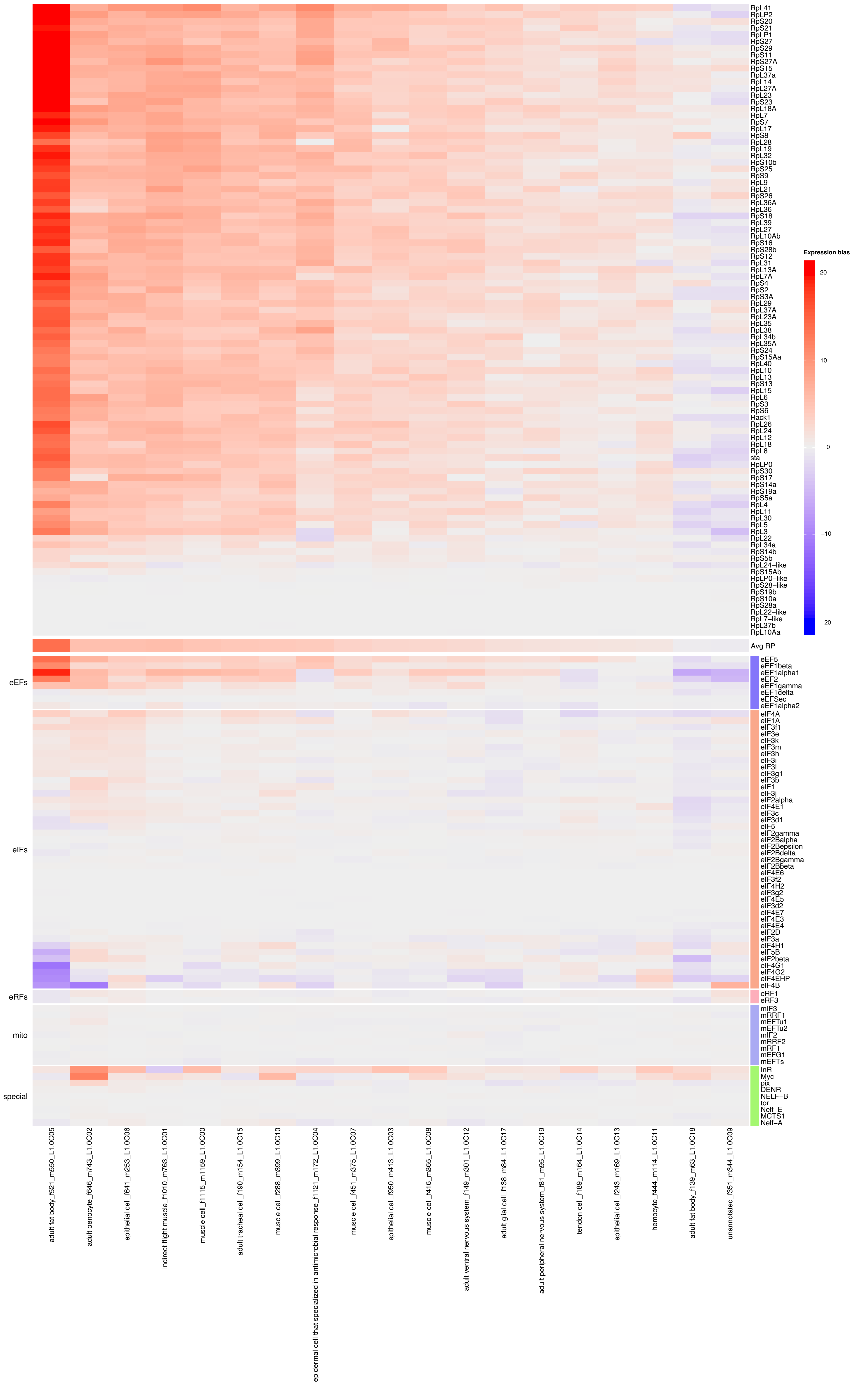

Supplement: Supplement 6 [file media-6.pdf]

Figure S7 - Expression Bias in Fatbody

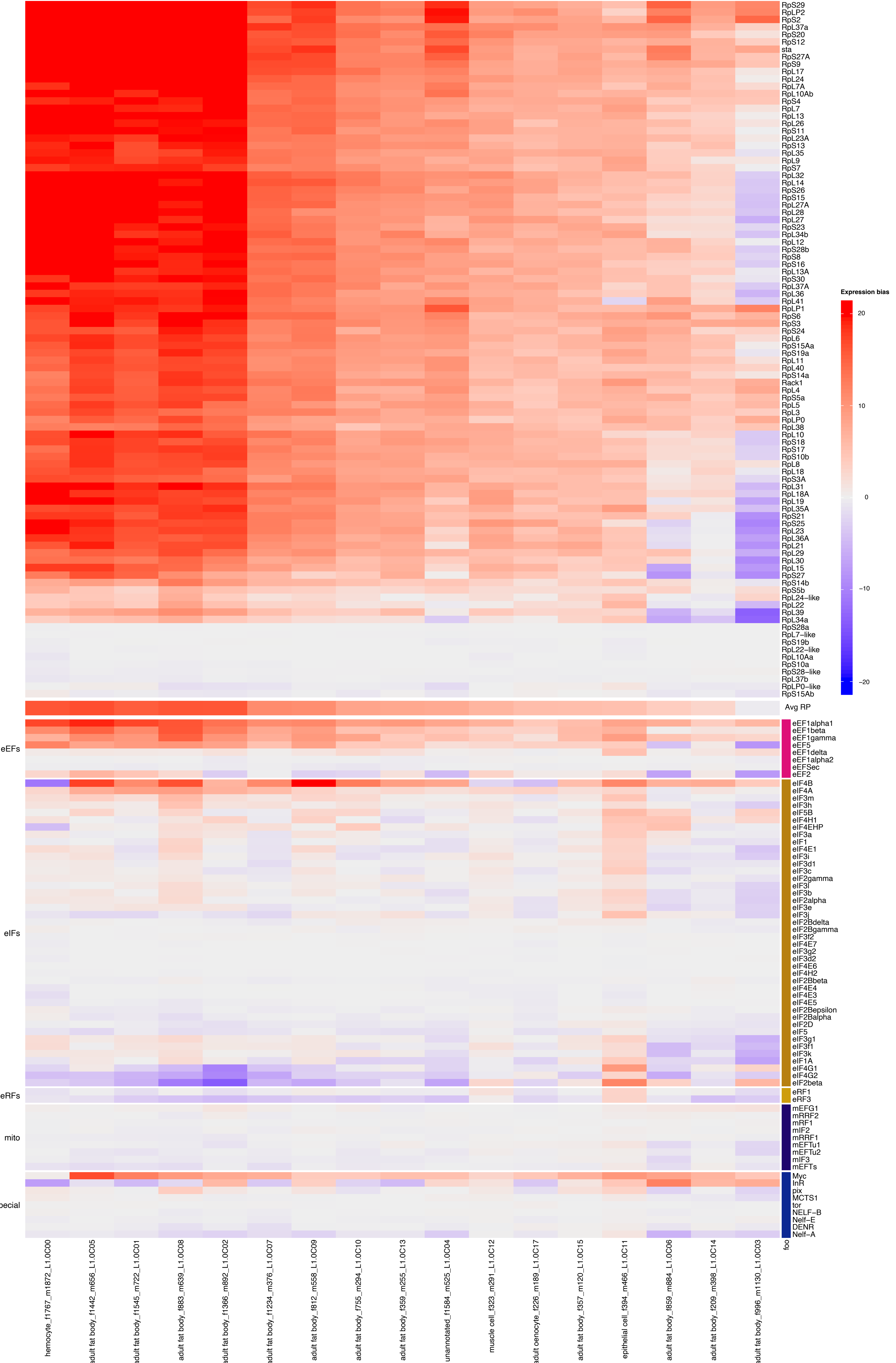

Supplement: Supplement 7 [file media-7.pdf]

Figure S8 - Expression Bias in Gut

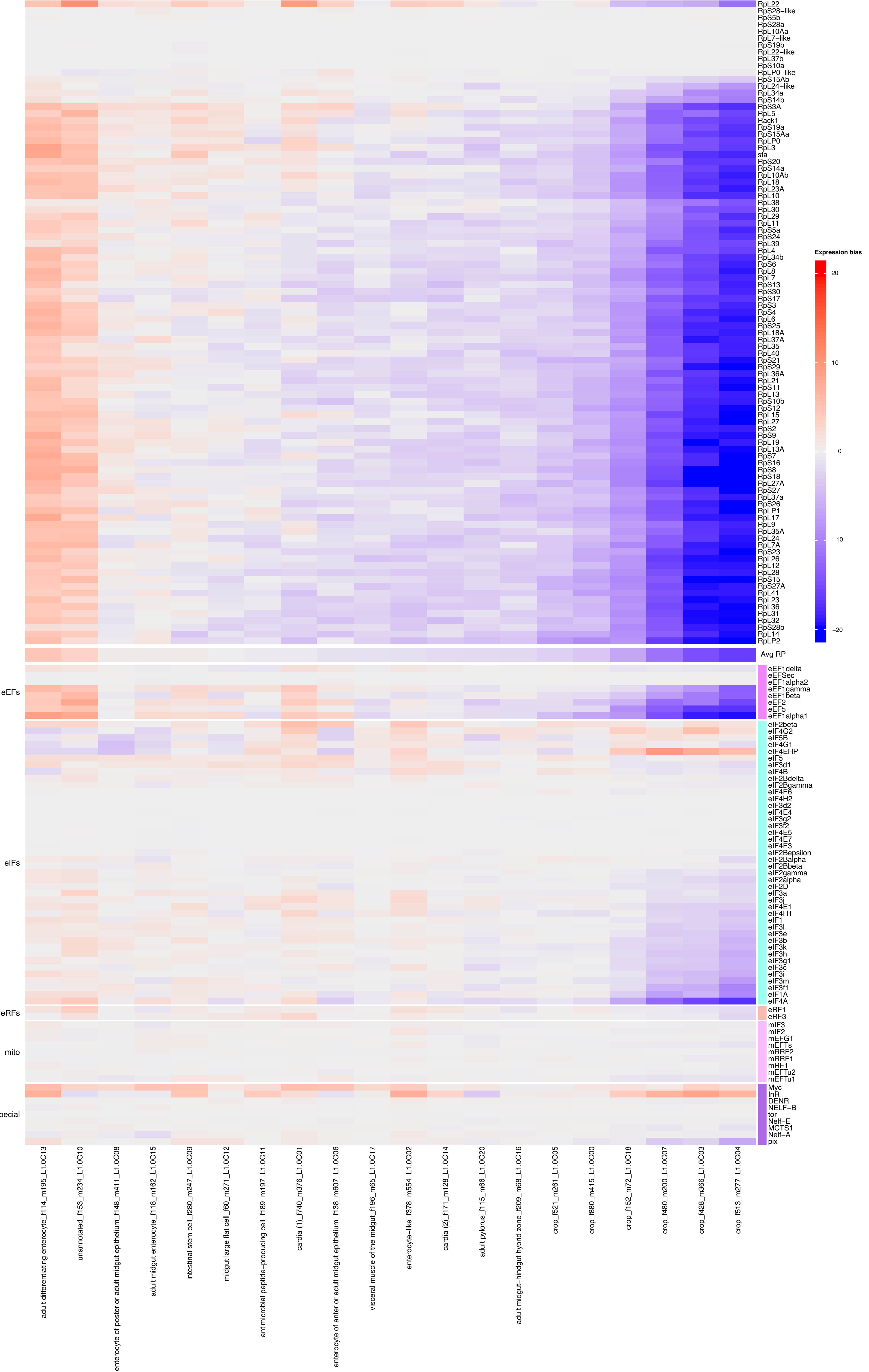

Supplement: Supplement 8 [file media-8.pdf]

Figure S9 - Expression Bias in Haltere

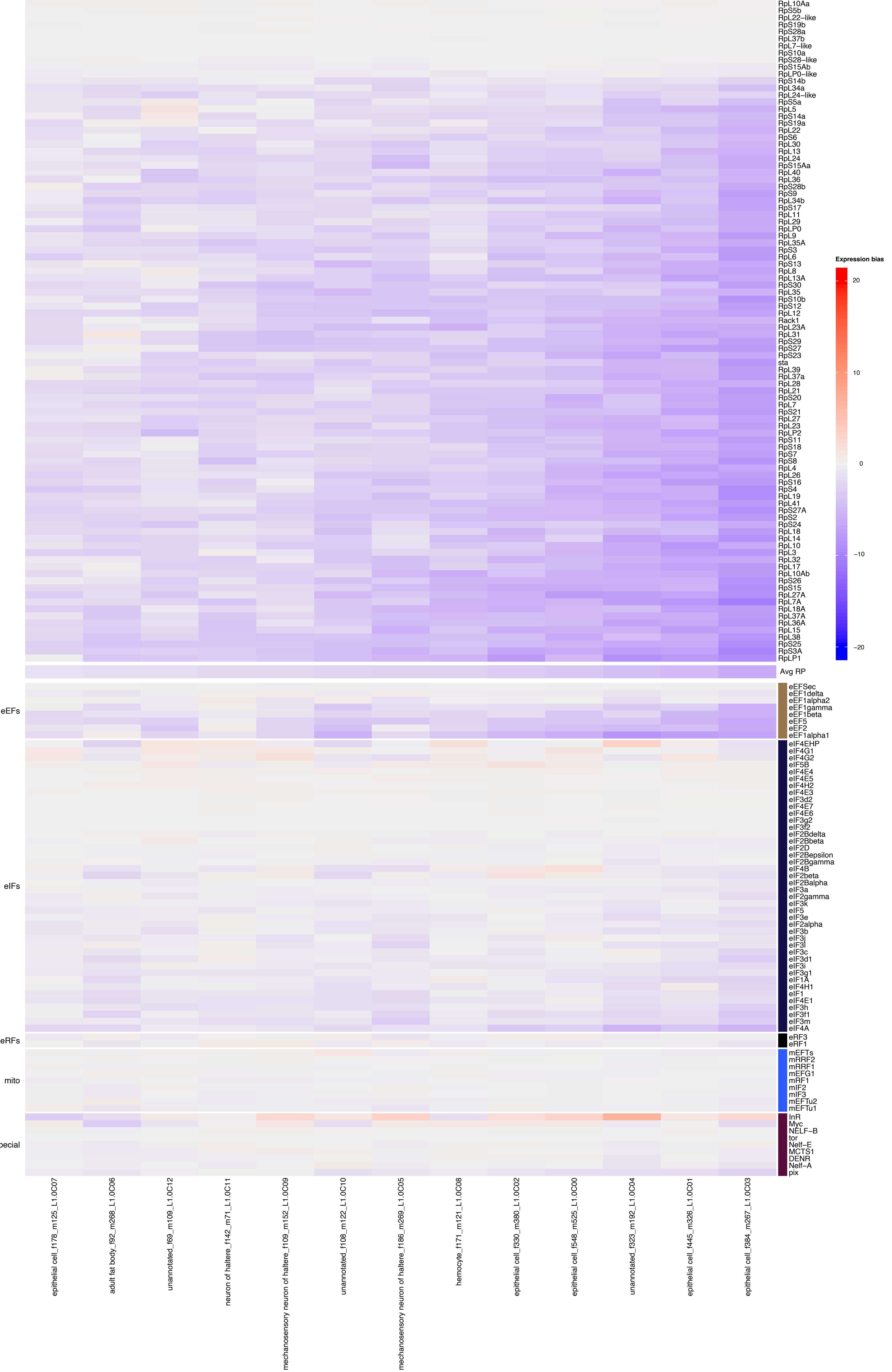

Supplement: Supplement 9 [file media-9.pdf]

Figure S10 - Expression Bias in Heart

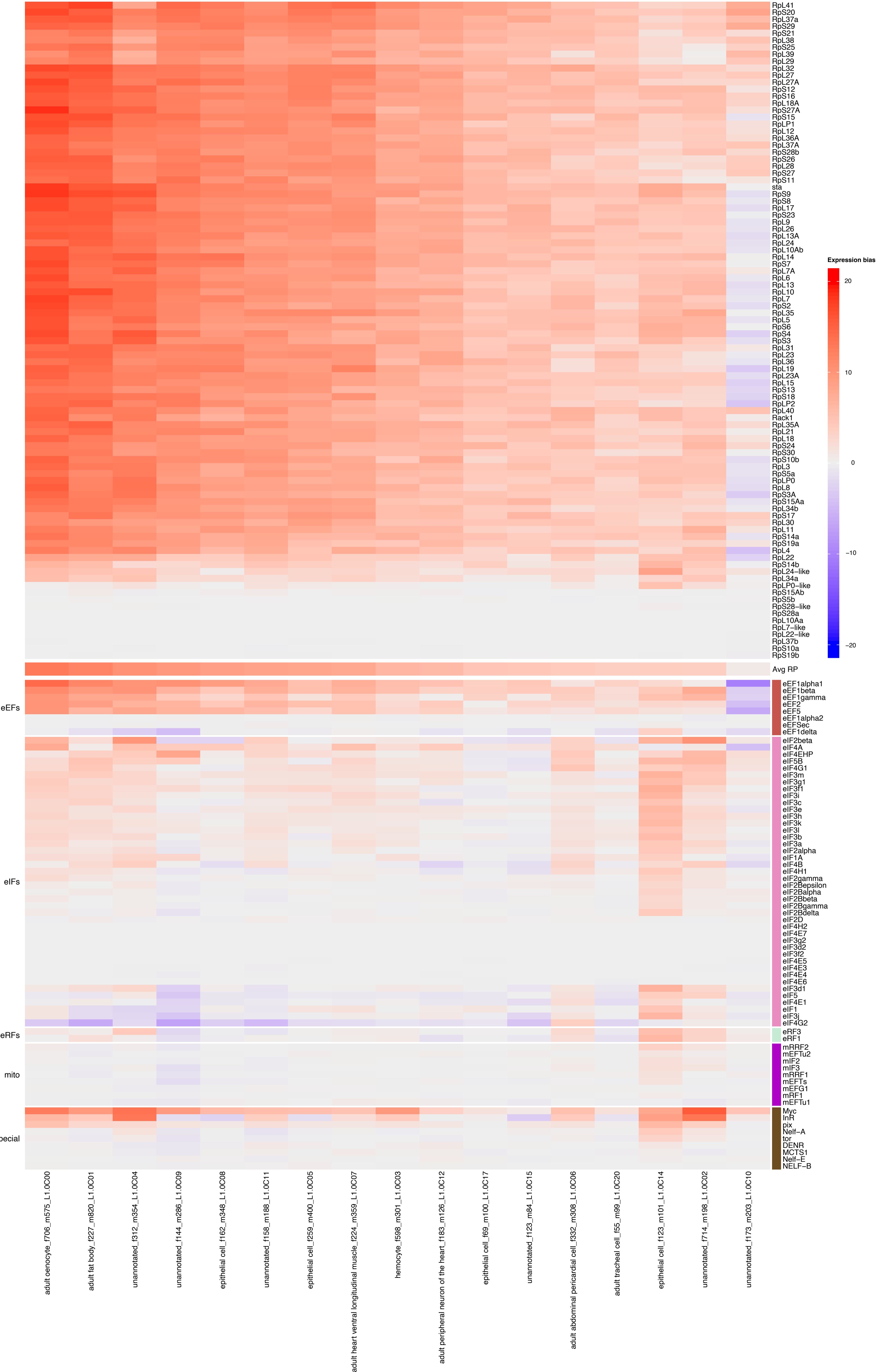

Supplement: Supplement 10 [file media-10.pdf]

Figure S11 - Expression Bias in Leg

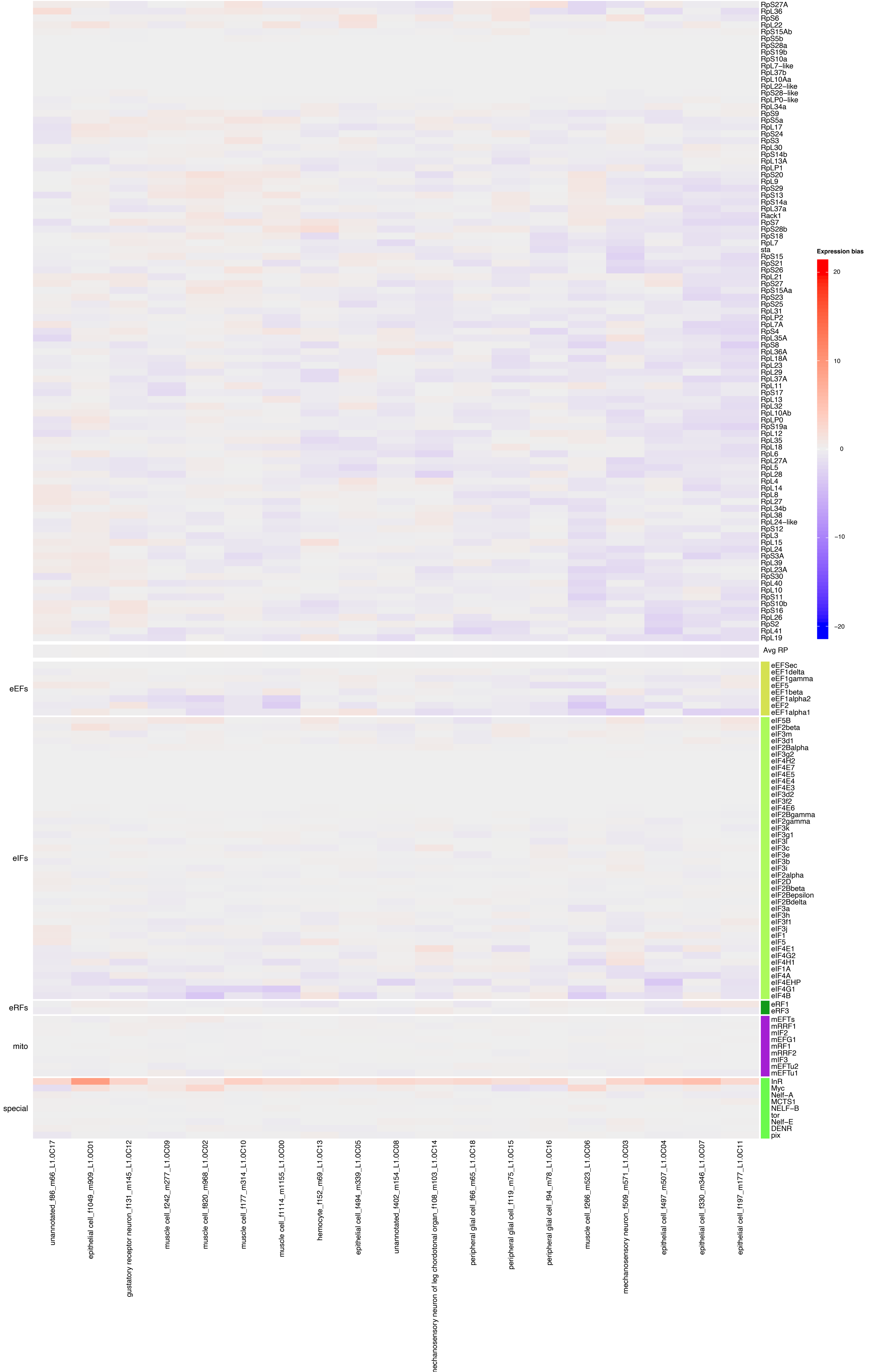

Supplement: Supplement 11 [file media-11.pdf]

Figure S12 - Expression Bias in Malpighian Tubule

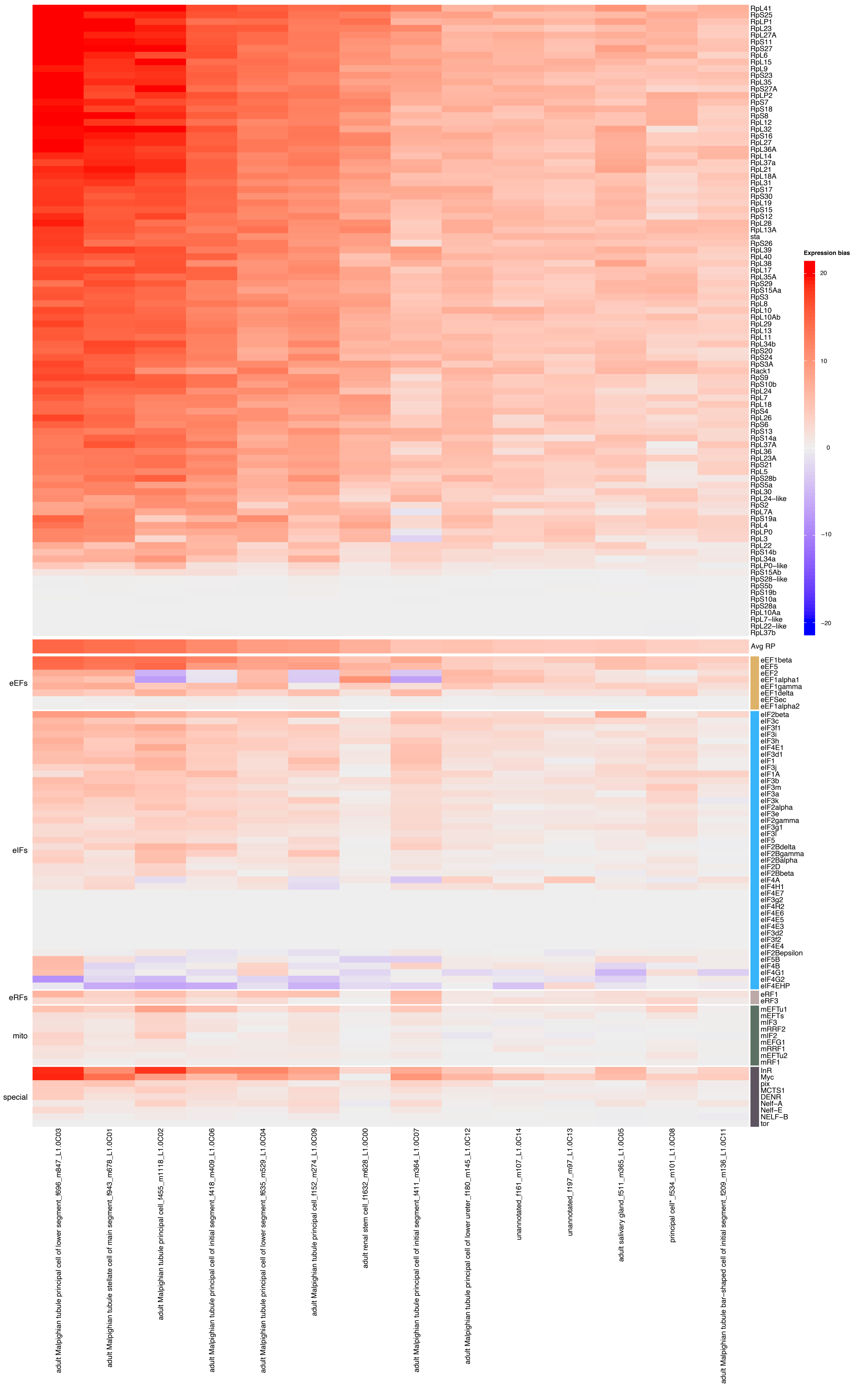

Supplement: Supplement 12 [file media-12.pdf]

Figure S13 - Expression Bias in Oenocyte

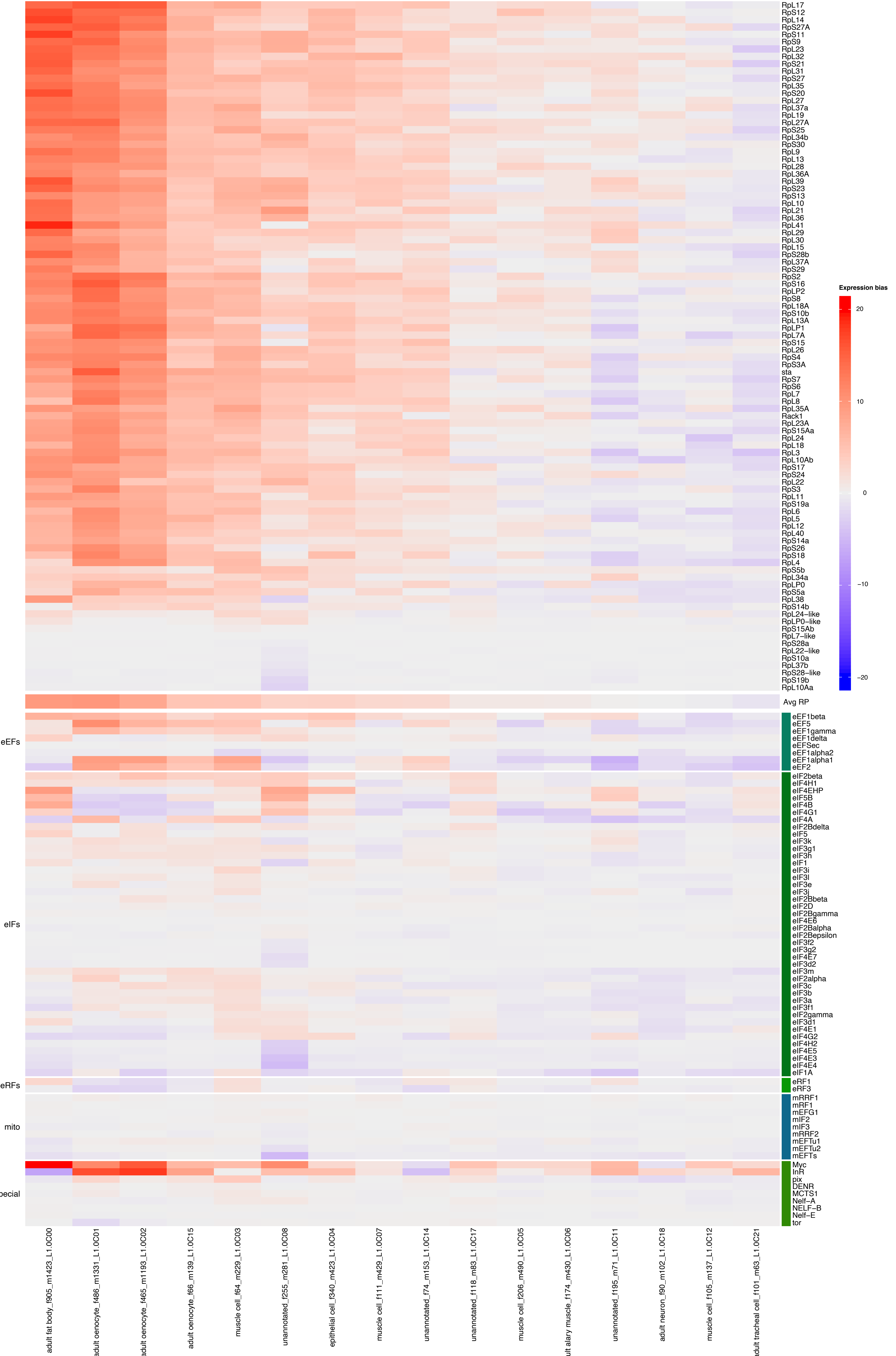

Supplement: Supplement 13 [file media-13.pdf]

Figure 14 - Expression Bias in Proboscis and Maxillary Palps

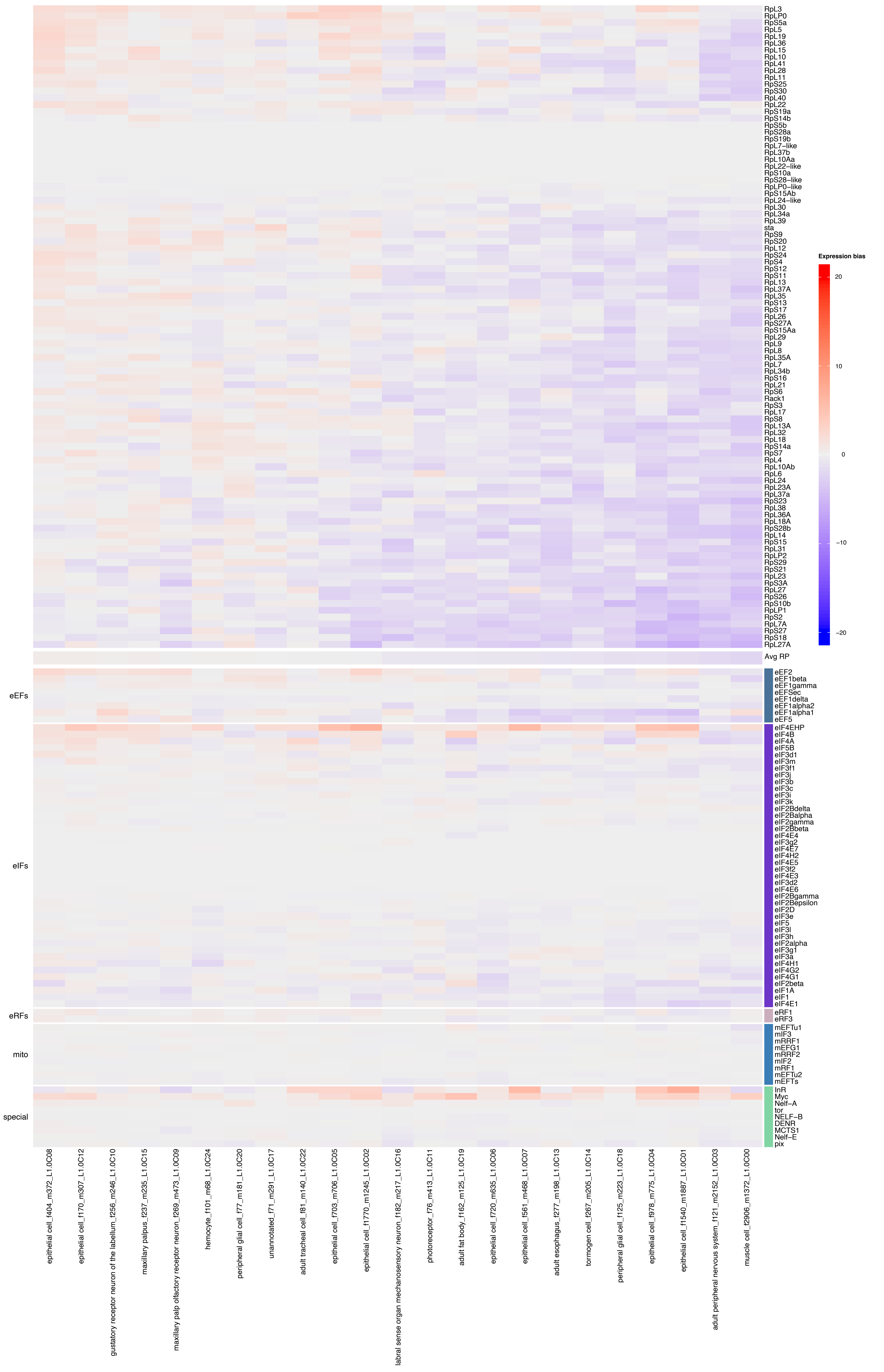

Supplement: Supplement 14 [file media-14.pdf]

Figure S15 - Expression Bias in Trachea

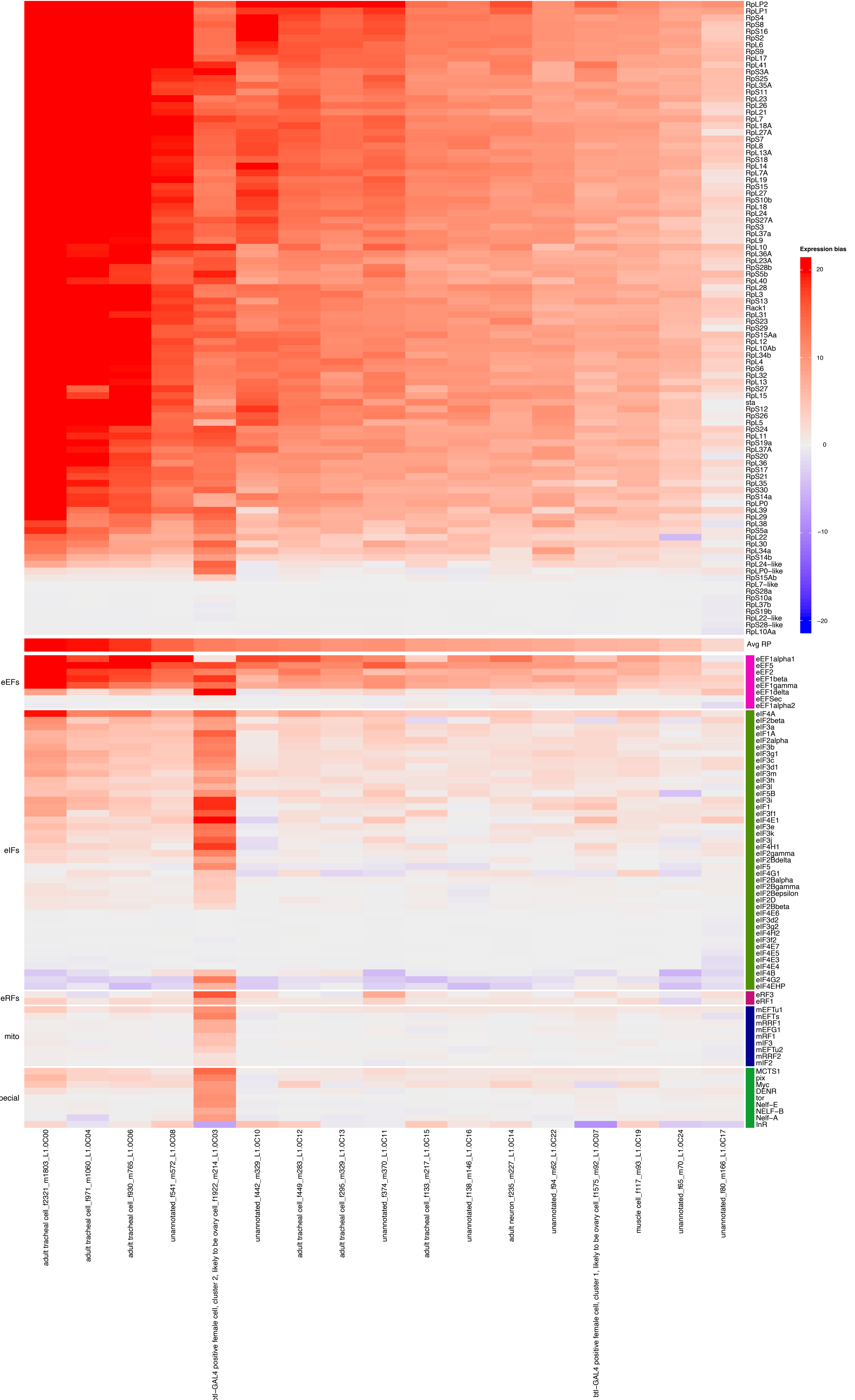

Supplement: Supplement 15 [file media-15.pdf]

Figure S16 - Expression Bias in Wing

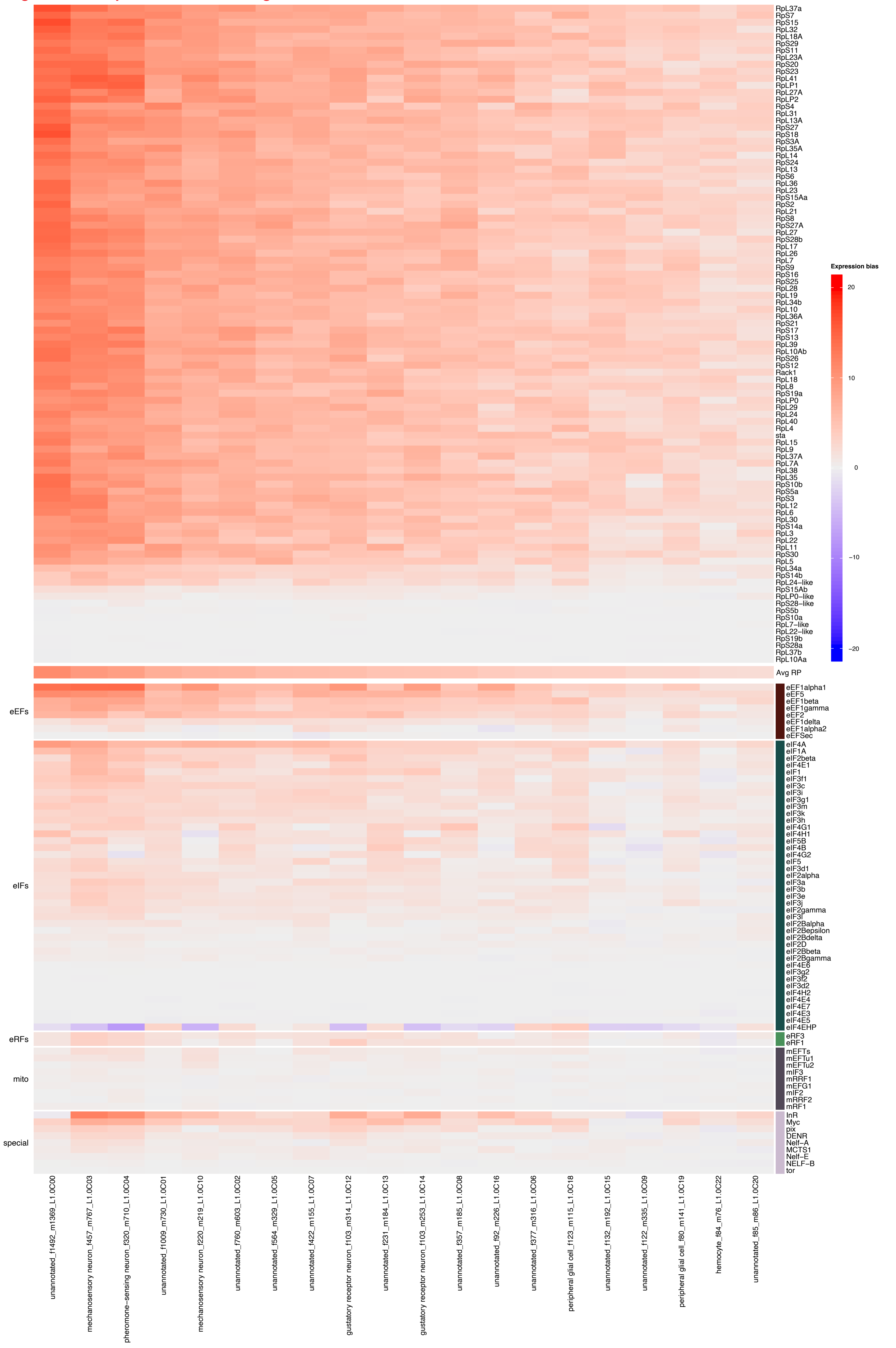

Supplement: Supplement 16 [file media-16.pdf]

# Figure S17

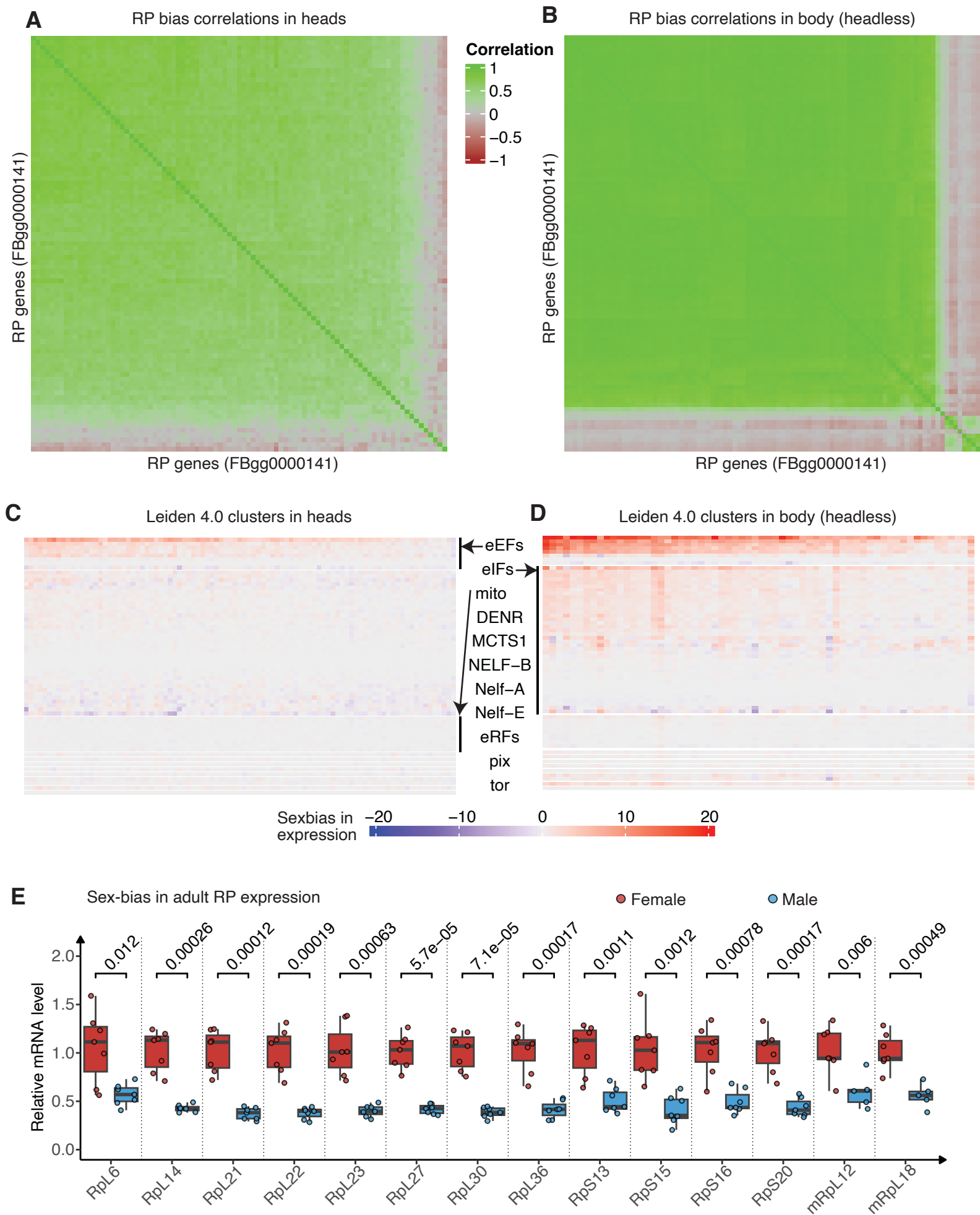

Supplement: Supplement 17 [file media-17.pdf]

# Figure S18

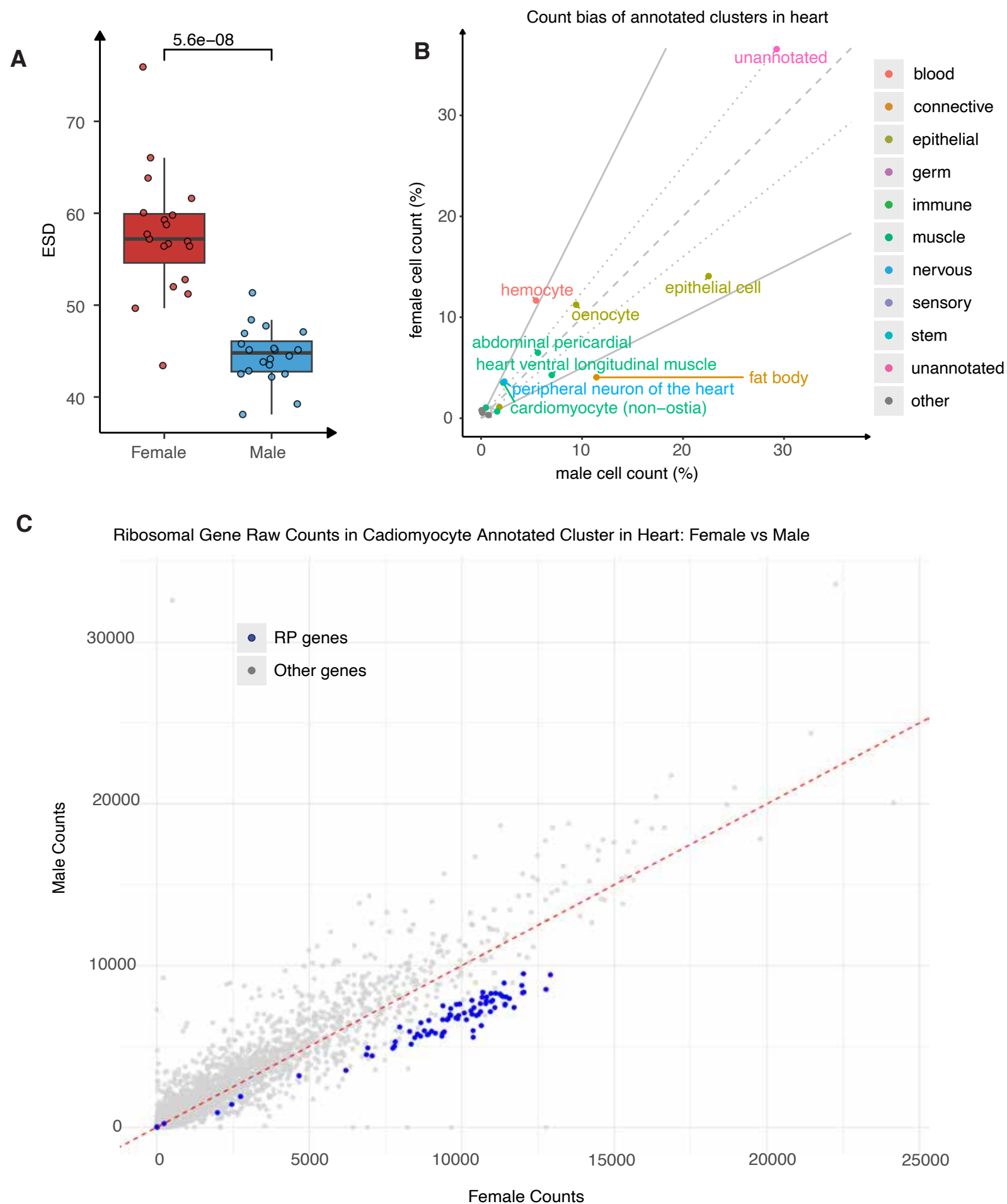

Supplement: Supplement 18 [file media-18.pdf]

# Figure S19

A

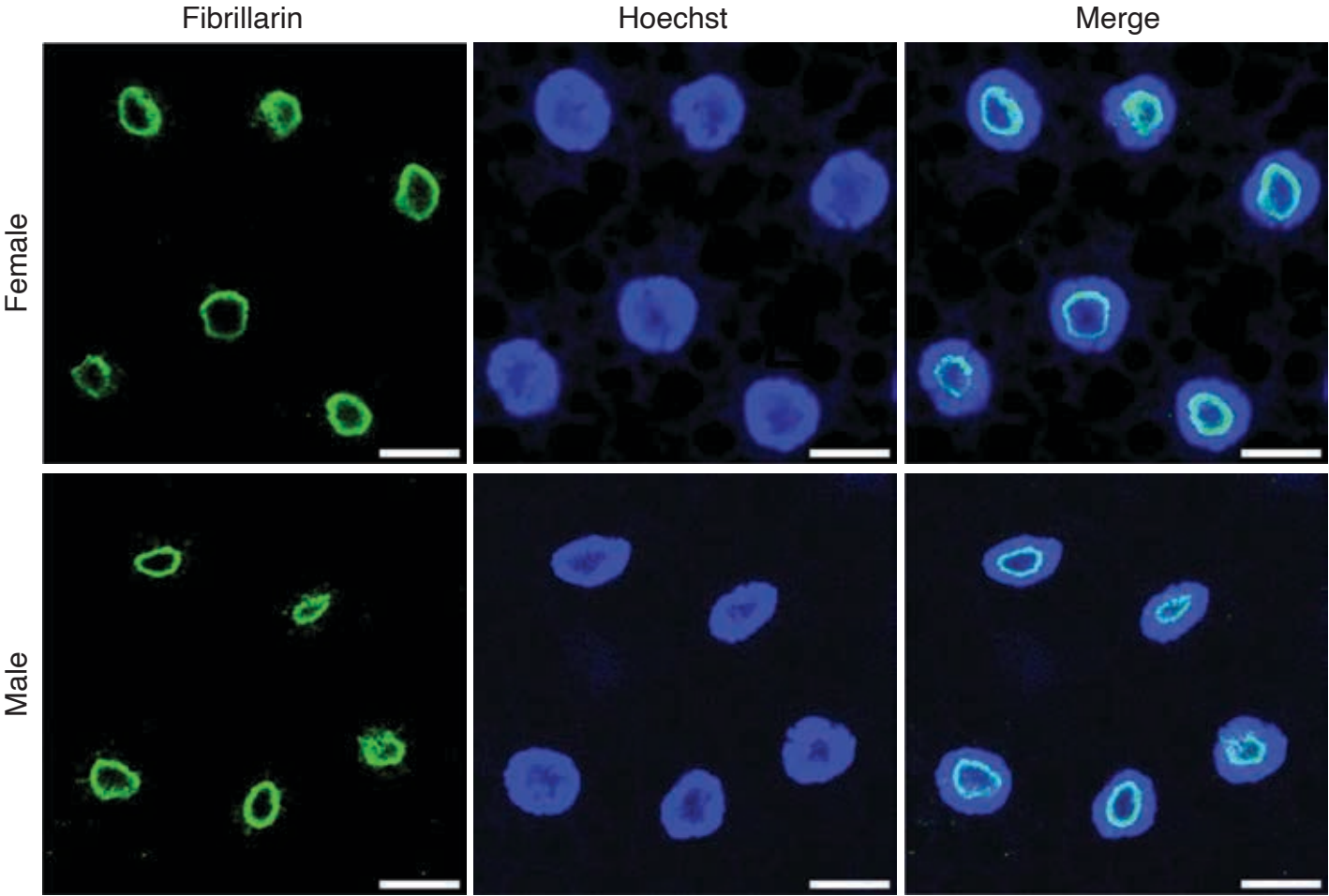

Supplement: Supplement 19 [file media-19.pdf]

**Figure S1 - Count Bias in first 6 dissected tissues**

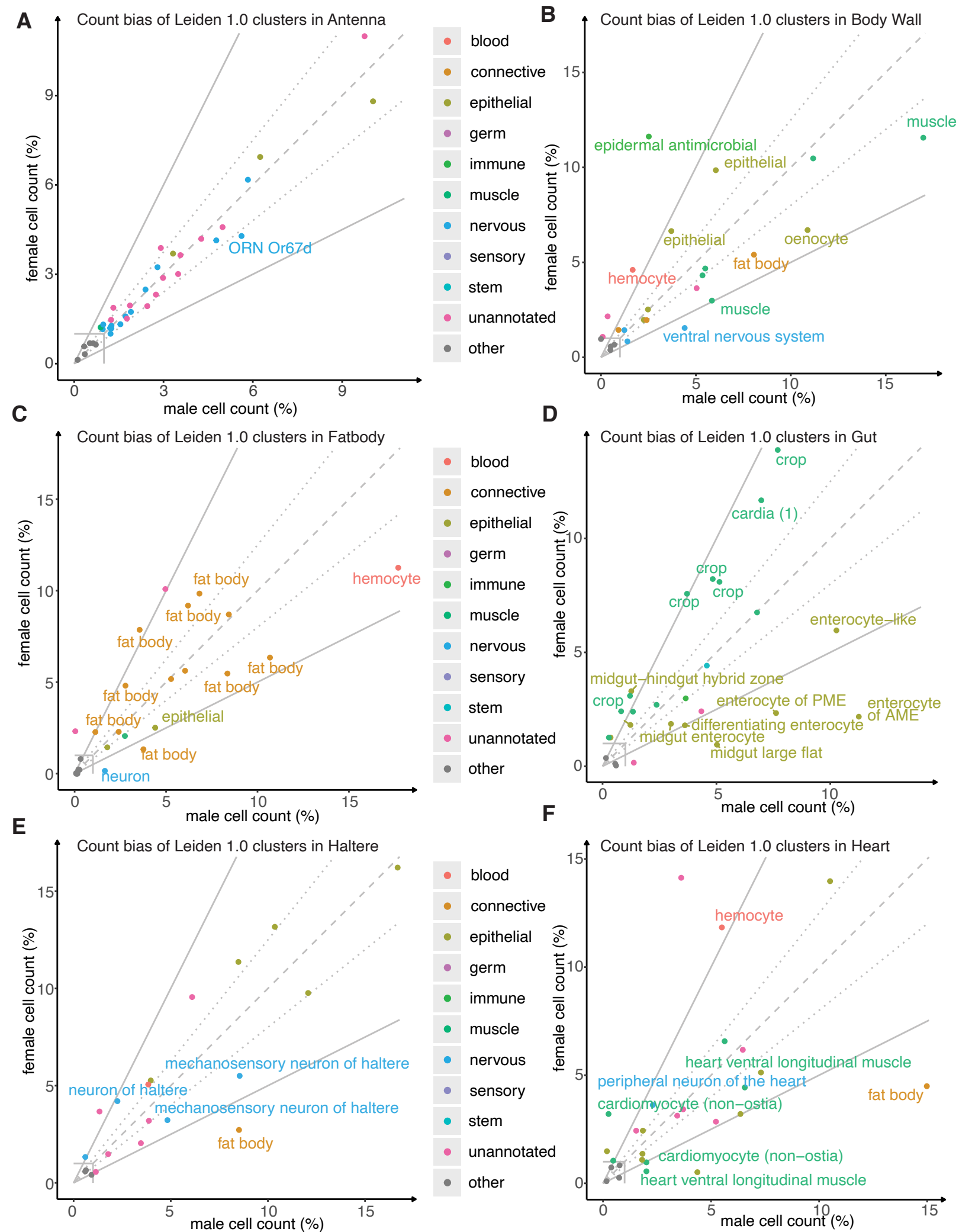

Supplement: Supplement 20 [file media-20.pdf]
