# Supplementary material for: Cell type specific allometry controls sex-differences in Drosophila body size
Source: bioRxiv. 2025 Aug 25:2025.08.25.671808. Preprint. [Version 1] doi: 10.1101/2025.08.25.671808 (PMC12407806; doi:10.1101/2025.08.25.671808)

Figure S2 - Count Bias in last 6 dissected tissues

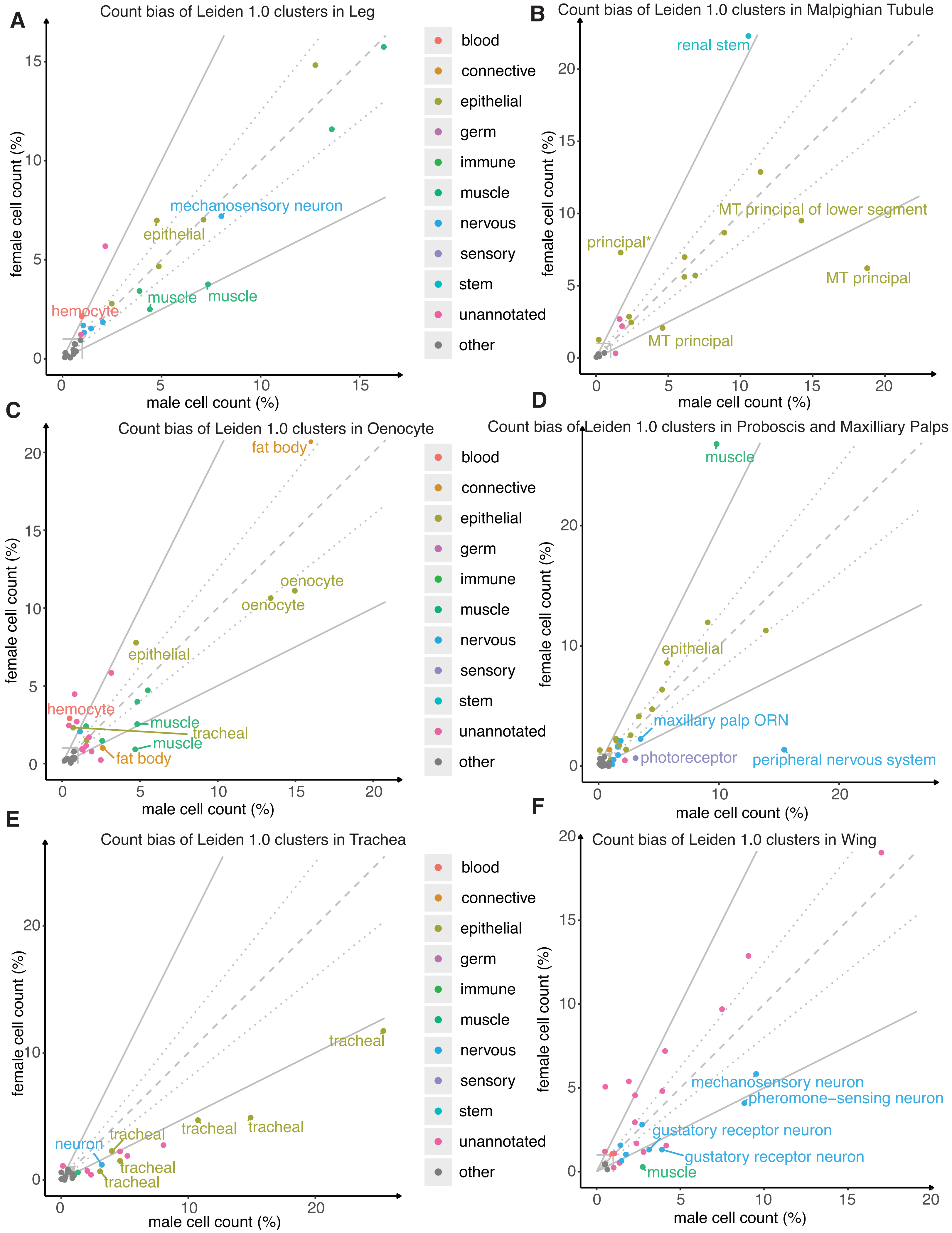

Supplement: Supplement 2 [file media-2.pdf]
